# Unraveling the Interplay of Physical-Chemical Factors Impacting the Carbonation Performance of Recycled Aggregate Concrete

**DOI:** 10.3390/ma16165692

**Published:** 2023-08-19

**Authors:** Carlos Pico-Cortés, Yury Villagrán-Zaccardi

**Affiliations:** 1 Multidisciplinary Training Laboratory for Technological Research (LEMIT), National Scientific and Technical Research Council (CONICET), Centro Científico Tecnológico La Plata, La Plata 1900, Argentina; carlospicocortes@conicet.gov.ar; 2 Sustainable Materials, Flemish Institute for Technological Research (VITO), Boeretang 200, 2400 Mol, Belgium

**Keywords:** carbonation, recycled aggregate concrete, ITZ, residual portlandite

## Abstract

Recycled aggregate concrete (RAC) includes recycled concrete aggregates (coarse and/or fine) as substitutes for natural aggregates as an approach to achieving a circular economy. Some concerns remain about its performance, including the carbonation resistance. The higher porosity of recycled concrete aggregates is logically a disadvantage, but the analysis must address many other factors. This paper provides an in-depth examination of recent advances in the carbonation performance of RAC. The emphasis is on factors that influence CO_2_ diffusion and the carbonation rate, e.g., the replacement ratio, source concrete quality, interfacial transition zone features, residual portlandite content, and porosity. The influences of previous treatments, combined action with supplementary cementitious materials, and loading conditions are also discussed. The replacement ratio has a significant impact on the carbonation performance of concrete, but it is also dependent on other factors. During carbonation, the physical effects of the porosity of the aggregate and the physical–chemical effects of the portlandite content in the adhered mortar are particularly important. The residual portlandite is especially significant because it is the primary hydration product responsible for the alkaline reserve for carbonation and the potential pozzolanic reaction, which are per se competing factors that determine the carbonation rate.

## 1. Introduction

Understanding the reactions occurring within concrete during its service life is crucial to predicting its durability upon interaction with the surrounding environment. Carbonation is a chemical reaction that primarily occurs between the calcium-bearing phases contained in cementitious materials (mostly portlandite) and carbon dioxide (CO_2_). This reaction happens in hardened concrete when CO_2_ enters from the environment, resulting in the formation of carbonates and reducing the pH from alkaline to neutral. The impact of carbonation is not derived from mere material science, but it has significant practical implications.

Studying carbonation is meaningful for two main reasons: durability and sustainability. Deeper knowledge of the carbonation process helps prevent the deterioration of reinforced concrete structures. When the pH decreases, the reinforcing steel becomes susceptible to corrosion [1], which can lead to a loss of design performance or the need for premature repair or demolition. From another perspective, carbonation plays a role in recapturing CO_2_ from the atmosphere and mitigating climate change to some extent. This forces us to address the issue of carbonation within the broader context of designing sustainable concrete structures.

Various strategies contribute to the sustainability of concrete. One approach involves extending the durability of the material to minimize waste and future clinker production. Recycled aggregate concrete (RAC) represents another sustainable strategy that uses construction and demolition waste (CDW) to restart the material’s life cycle. Maybe even more important is reducing the clinker factor by substituting clinker with supplementary cementitious materials, along with the consideration of residual carbon capture capacity. All these strategies are linked to the carbonation process in various ways. The connection becomes clearer after considering the reactions occurring in the carbonation of concrete.

The carbonation process of concrete consists of several reactions. Gaseous CO_2_ dissolves in an alkaline aqueous medium (1), which favors the formation of insoluble carbonates [2]. Carbonates are formed from the reaction of calcium ions in solution (2) or portlandite crystals (3) [3] with the CO_2_. Additionally, other alkalis can react with the CO_2_ to form sodium and potassium carbonates, which are more soluble but still lower the pH. Finally, carbonation consumes calcium from other compounds in hardened concrete (e.g., hydrated calcium silicate, C-S-H (4), and hydrated aluminate (5)). The alkaline reserve decreases, causing the pH to drop and increasing the corrosion risk for the structural steel.

(1) $${\mathrm{CO}}_{2}\left(g\right)+H_{2}O\left(l\right)\to {\mathrm{CO}}_{3}{}^{2-}\left(\mathrm{ac}\right)+H_{2}O\left(l\right)$$

(2) $${\mathrm{Ca}}^{2+}\left(\mathrm{ac}\right)+{\mathrm{CO}}_{3}{}^{2-}\left(\mathrm{ac}\right)\to {\mathrm{CaCO}}_{3}\left(s\right)$$

(3) $$\mathrm{Ca}{\left(\mathrm{OH}\right)}_{2}\left(s\right)+{\mathrm{CO}}_{2}\left(g\right)\to {\mathrm{CaCO}}_{3}\left(s\right)+H_{2}O\left(l\right)$$

(4) $$\mathrm{xCaO}\left(s\right)\cdot {\mathrm{SiO}}_{2}\left(s\right)\cdot {\mathrm{yH}}_{2}O\left(s\right)+{\mathrm{xCO}}_{2}\left(g\right)\;\to {\mathrm{xCaCO}}_{3}\left(s\right)+{\mathrm{SiO}}_{2}\left(s\right)\cdot {\mathrm{tH}}_{2}O\left(s\right)+\left(y-t\right)H_{2}O\left(l\right)$$

(5) $$4\mathrm{CaO}\left(s\right)\cdot {\mathrm{Al}}_{2}O_{3}\left(s\right)\cdot 13H_{2}O\left(l\right)+4{\mathrm{CO}}_{2}\left(g\right)\;\to 4{\mathrm{CaCO}}_{3}\left(s\right)+2\mathrm{Al}{\left(\mathrm{OH}\right)}_{3}\left(s\right)+10H_{2}O\left(l\right)$$

The carbonation rate, kc, refers to the rate of progression of the depth or carbonation front (e). It is traditionally modeled according to a particular solution of Fick’s second law (6). Tuutti [1], one of the pioneers in the application of this traditional approach, suggested the value of 0.5 for the exponent in the equation, eventually changing to lower values under certain conditions. Predictive models have advanced to account for more variables, some of which include RAC-specific features.

(6) $$e=kc\cdot \;t^{0.5}$$

RAC undergoes carbonation with its own intricacies that make it different from conventional concrete. In contrast to conventional concrete, RAC combines various additional material phases that impact carbonation performance. From a chemistry point of view, RAC’s carbonation mechanism is impacted by a balance left over from earlier carbonation or even specific external attacks during its previous use(s) [4]. From a physical point of view, the particular porous structure generated by the adhered mortar incorporates an additional porous phase and interfacial transition zone (ITZ) for all the involved transport processes [5]. The adhered mortar is the amount of matrix phase that remains in the recycled concrete aggregate (further indicated as RCA), which is mostly bonded to natural aggregate particles but can also be found as independent particles. The volume of adhered mortar and the texture influence the generation of particular ITZs that are differentiated from those formed by the natural aggregate. The only alternative that still allows for prognosis analysis is directly measuring the carbonation rate in the new RAC. This is due to the complexity of the reactions in these attacks as well as the variation in the alkaline reserve.

Building on this basic assessment, the primary objective of this review is to provide a comprehensive analysis of the factors involved in the carbonation resistance of concrete. This encompasses the features of the recycled concrete aggregates, enhancement processing, and mix design. The scope of this review is to provide the necessary input for the effective prediction of the front depth (relevant for durability), which depends on physicochemical and experimental factors. Subsequent sections discuss the main recent developments in our understanding of the unique characteristics of RAC carbonation.

## 2. Influencing Factors in Carbonation Rate

### 2.1. Effects of the Replacement Rate of NA by RCA

**Figure 1:**
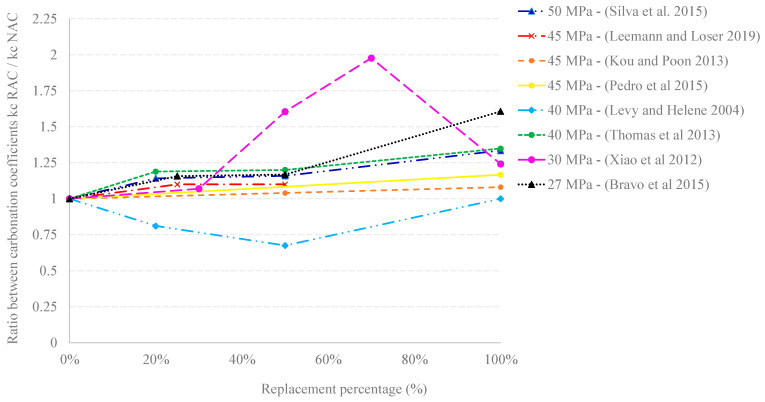
Effect of the replacement rate of NA by RCA on the ratio between the carbonation rate of RAC of different characteristic strengths and the carbonation rate of NAC. Data from: Kou and Poon 2013 [6], Bravo et al., 2015 [7], Pedro et al., 2015 [8], Thomas et al., 2013 [9], Silva et al., 2015 [10], Leemann and Loser 2019 [11], Xiao et al., 2012 [12], Levy and Helene 2004 [13].

With a variety of outcomes, numerous investigations have sought to establish the impact of the rate of replacement of NA (natural aggregate) by RCA on the carbonation rate of concrete. This variability can be partly explained by the various test methodologies proposed worldwide and the various exposure periods and CO_2_ concentrations, which can range from natural to accelerated levels. Figure 1 shows the relationship in the literature between the carbonation coefficient of RAC and the carbonation coefficient of its respective control concrete. Some investigations have approximated natural long-term tests such as those performed by [6] using accelerated tests [7,8,9].

The literature indicates that, in general, RCA increases the carbonation rate in concrete [10,14,15]. For instance, [10] reported that a 100% RCA content leads to an increase of between 20 and 100% in the carbonation rate, depending on the RCA features. More recently, encouraging results for the RAC carbonation rate were shown for substitution percentages between 25% and 50%. More recently, more encouraging results for the RAC accelerated carbonation rate showed increases in the carbonation coefficient (kc) of only 10% for replacement ratios between 25% and 50% [11]. This study reported the sizable impact of the cement type on the overall effect. To ensure buffering capacity against CO_2_ ingress into concrete, it is crucial to use cements that lead to significant amounts of reactive CaO in the hydration products.

Such affected performance of RAC is mainly attributed to the higher porosity. However, the impact is not always directly proportional to the substitution of NA by RCA [16], due to additional factors specific to RAC such as buffer capacity. Particular studies on natural carbonation [12] reported 70% replacement as the percentage for which a change in the trend occurred. As RCA content increases, an increase in the carbonation rate (mainly influenced by physical aspects) is observed for lower replacement ratios, but a stabilization is noted at 70% replacement, and decreased carbonation rates are reported for higher RCA contents. This threshold value in RCA content was explained by a shift towards dominant chemical effects above 70% replacement, where the buffer capacity of the adhered mortar begins to dominate the net effect. Results found by [13] go even further and report RAC having a favorable ability to counteract carbonation in comparison with NAC, especially at replacement ratios between 20% and 50%. However, the detailed analysis of the mixes in that report reveals a greater cement content in the RAC mixes to achieve similar fresh performance, and the improvement in performance can be attributed to the richer matrix instead of the aggregate content. The mix design method and the manner in which the effective water/cement ratio is affected by the incorporation of RCA are key to the outcome of this comparative analysis [17].

It is intriguing to examine the possibility of confirming such an optimum for the RCA content in studies that evaluate the entire range between 0% and 100% replacement in terms of carbonation performance. The critical RCA content could then be defined as the RCA content at which the chemical factor begins to dominate carbonation performance. This factor will depend on the specific features of the RCA. The buffer capacity would be associated with the quality of the RCA and its content of reactive CaO, mainly defined by the cement type used in the source concrete.

### 2.2. Origin of RCA

The RCA origin has produced a range of outcomes for RAC’s carbonation. Performance can vary depending on factors such as the type of natural aggregate used in the original concrete, the features of the original mix, the past conditions of service, and the crushing and sorting procedures used during recycling. For instance, concretes with two types of RCA were evaluated in [18]. The first type was from the crushing of concrete specimens manufactured in the laboratory, and the second was from demolition in a power plant. The findings showed that for the first type, the durability indices of RAC were comparable to those of NAC. For the second type, however, there was an increase in the accelerated carbonation depth (+23%) at 26 weeks of age. Similar to this, Otsuki et al. [15] showed that RAC made with a high-quality RCA coming from a 51.7 MPa concrete demonstrated a higher carbonation depth by only 4.8% with respect to NAC. Still, it was found that even for other sources of RCA from concretes with the same original strength, the results could vary depending on the amount of adhered mortar.

Regarding the type of natural aggregate used in the source concrete, the differences between siliceous gravel, crushed granite, crushed basalt, and crushed quartzite aggregates have shown an impact on the transport properties of concrete. Such properties can be used to infer a similar trend for the carbonation rate. For instance, the capillary absorption coefficient is an index considered by durability guidelines, including for the use of RA [4,19]. It was determined in [20] that, at higher RCA contents, siliceous gravel and quartzite aggregates affected water penetration in the RAC to a lesser extent, despite being more porous aggregates than granite and basalt. Such behavior was attributed to the improvement of aggregate-mortar interfaces in the RCA with respect to the corresponding NA. However, these same types of aggregates presented higher capillary suction than the other two aggregate types at higher replacement percentages. This is due to the fact that this property is more influenced by the porosity and roughness of the NA.

### 2.3. Adhered Mortar and Quality Parameters

First and foremost, the influence of adhered mortar on the performance of RAC leads to higher permeability. The impact of the content of adhered mortar and its quality is mainly evidenced by the value of water absorption [15]. The results showed a trend of approximately 1% increase in the absorption value for each 10% of additional adhered mortar in RCA coming from concrete with strengths between 51.7 and 68.5 MPa. When the absorption of the aggregate was checked after removing the mortar, it was found to be only 0.15% above the value for the corresponding NA in the same study.

The content of adhered mortar may lead to changes in the effective water/binder ratio (*w_eff_/b*) of the new mix. The *w_eff_/b* differs from the commonly used water/binder ratio (*w/b*) in that the former only considers the amount of free water in the fresh mix rather than the total amount of mixing water. The incorrect consideration of the water absorption of RCA frequently leads to significant differences between the two concepts. This has an impact on the performance of RAC in both fresh and hardened states. In this regard, it has been argued that carbonation resistance could be improved with double mixing procedures that favor pre-saturation of the RCA before adding the rest of the constituents. In [15], it was determined that double mixing resulted in a 12.3% lower carbonation rate with respect to single mixing. This trend was mostly demonstrated for RAC with high *w/b* (0.55). Mixes with very low *w/b* (*w/b* = 0.25, with corresponding values of compressive strength greater than 85 MPa) showed a minimal impact of the RCAs on the carbonation resistance regardless of the adhered mortar. Additionally, in [21], it is demonstrated that an optimized mixing procedure can reduce the influence of the attached mortar and, overall, the carbonation depth of RAC.

The effects of the adhered mortar on carbonation may be somehow different from the effects on properties involving purely physical aspects due to the associated buffer effect. The results of [12,16], where RCA contents above 70% (adhered mortar of 40% wt.) showed carbonation rates relatively independent of the replacement ratio, can be explained by the simultaneous double effect of increased porosity and increased alkaline reserve. The heterogeneity of the carbonation depth that tends to occur between different zones of the RAC is another effect of the adhered mortar. This is easily visible after spraying with phenolphthalein when the profile of the carbonation front deviates around RCA particles. Something less visible but revealed through chemical analyses is that the effect on the degree of carbonation (degree of reaction of carbonatable phases in concrete) can vary with respect to the NAC [22]. This key parameter is essential to both calculate the CO_2_ uptake by the carbonation of the material and model the progress of carbonation [23]. Such a contrast with NAC suggests the need for going beyond the practical assessment of carbonation with a pH indicator and coupling it with the assessment of the degree of carbonation for the estimation of the CO_2_ uptake of RAC [22].

The quality of the RCA, mainly related to the content of adhered mortar and its specific porosity, can largely determine the effects of the replacement rate. Water absorption and crushing resistance can be used to assess quality [18]. Water absorption reflects the porosity of RA, which, in association with the replacement ratio, affects the strength, durability, and amount of compensation water needed in concrete. Care must be taken to adjust the amount of mixing water so that *w_eff_/b* is not affected by the inclusion of RCA. Varying correction criteria can lead to different conclusions about the impact of RCA. Such precaution is not always addressed properly in the literature, and a fictional magnification of the impact of RCA is sometimes reported [24]. A study by Bravo et al. [7] depicts the influence of RCA quality on carbonation resistance. They evaluated the carbonation performance of RAC with various RCAs from several Portuguese plants. For a 100% RCA content, the authors observed increases of between 22.2% and 182.4% in the carbonation coefficient with respect to NAC after 28 days of curing. The significant variability was primarily associated with the quality of recycled aggregate provided by each plant. The content of clay products (bricks, tiles) in RCA worsened the performance due to their higher porosity compared to waste concrete. Consistent with this, Soares et al. [25] suggested that when the quality of RCA is similar to that of NA, especially in terms of density, wear resistance, and absorption, the variability to be expected is lower. On such a basis, they reported negligible differences between RAC and NAC of between 0.2 mm and 1.2 mm in the accelerated carbonation depth measured at 7, 28, 56, and 91 days.

Other parameters that also qualify RCAs are particle size distribution, flakiness and elongation, resistance to sodium sulfate, dust content, and chloride or sulfate content [26]. The correlation between these factors and the carbonation performance of RAC is not yet well reported in the literature.

### 2.4. Effects of External Loads

Internal stresses caused by external compressive or tensile loads can change the carbonation rate. For very low axial compressive loads, the carbonation rate may decrease, while for intermediate to high compressive or tensile loads, it may increase considerably. According to [27], carbonation decreases when the load level (applied stress/ultimate stress) in compression is lower than 0.2 and increases when it is higher than 0.2.

In [12], carbonation tests of RAC under external tensile loads were carried out. The relative carbonation depth increased with increasing tensile stress. This increase was 70%, compared to the action of an external bending load with experimental stress levels close to its estimated tensile strength. For a stress level of 0.6 times the tensile strength, it was barely 20%. These results were attributed to microcracking. In addition, in the presence of external loads, it was found that the carbonation rate of the concrete increased with the recycled content for RCA contents lower than 70% [12]. This threshold reported for unloaded samples maintains under load, and no significant difference resulting from further increasing RCA over 70% was reported.

Loading leads to cracks in structural elements. These may have crack widths that are sometimes admissible and sometimes non-compliant, but no matter the width, the presence of cracks favors carbonation in conventional concrete and RAC. In [28], it is shown that even a mere 0.05 mm crack doubles the carbonation depth compared to uncracked RAC samples. This impact was lower for NAC than for RAC, as at the same crack width, 15% less carbonation was reported for the former. The impact is not totally local, as the zone affected by the presence of cracks can be as wide as 10 mm.

Concerning the connection between carbonation and structural performance, it has been reported that carbonation has noticeable effects on the elasticity of concrete specimens [29,30]. This implies a cross-link between internal stresses and carbonation. The presence of high-level stresses facilitates carbonation, and at the same time, the progression of carbonation increases the modulus of elasticity of concrete and the peak stress when tested to failure under compression.

### 2.5. Interfacial Transition Zones (ITZ)

**Figure 2:**
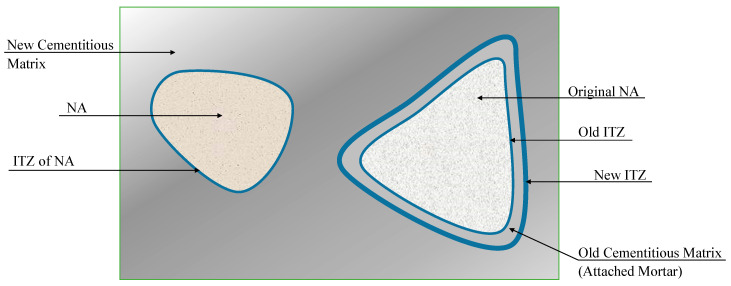
Schematic diagram of zones that make up the RAC, including several ITZs.

The combination of RCA and NA in new concrete defines seven zones in RAC (Figure 2): original aggregate, old cementitious matrix (or adhered mortar when not removed), old ITZ (between the old matrix and the original aggregate), new cementitious matrix, new ITZ (between the new matrix and the adhered mortar), NA, and NA-corresponding ITZ with the new matrix. The microstructure and other features of each zone determine the interactions and mass transport of the system.

It is well known that the new phases introduced with the inclusion of RCA in concrete influence the durable properties of RAC [15]. As for the characterization of the ITZ, Vickers microhardness can profile the porosity across the interfaces to reveal the effects on it produced by, for instance, the mixing method, the *w/c*, and the strength level of the original concrete. A consistent description of the microstructure of the ITZ was created by Poon et al. [31] through scanning electron microscopy (SEM) images that detected the loose and porous hydrates in the RCA ITZ in contrast to the denser hydrates presented in the NA ITZ.

The quality of the ITZ is highly affected by the saturation state of the RCA at the moment of producing the mix. This is an aspect not sufficiently considered in the literature, which tends to address mostly the effects of the proportioning and much less the effects of the processing. When RCAs are incorporated unsaturated, they will absorb water during mixing and cause significant effects concerning the ITZ. This is a stronger bond between the new cement matrix and the RCA at early ages. Poon et al. [32] argue this is due to the water migration towards RCA particles in the fresh mix. When RCA are pre-saturated, free water remains on the surface of the particles when incorporated in the mix, which may cause a detriment to the properties of concrete in the hardened state [33]. It has been proposed to lengthen the times of a single mixing, taking into account the water absorption rate of the RCA [34]. However, to favor the microstructure, it is generally preferred to incorporate RCA with a previous wet mixing but without reaching saturation to avoid damaging the ITZ. The pre-wetting may have the advantage of favoring the hydration of cement at the ITZ [35], which is especially important in concrete, for which the humid curing of on-site RAC may be compromised by the external environment. In any case, it is important to ensure that the correction and mixing methods do not affect the *w_eff_/b* of the mix [24,36].

In terms of carbonation, more porous ITZ facilitates the penetration of CO_2_ through the microstructure. In the particular case of RAC, with two kinds of matrices and three kinds of ITZs, the contrast between phases can cause inhomogeneities in the carbonation profile [37,38].

### 2.6. Influence of Crushing

**Figure 3:**
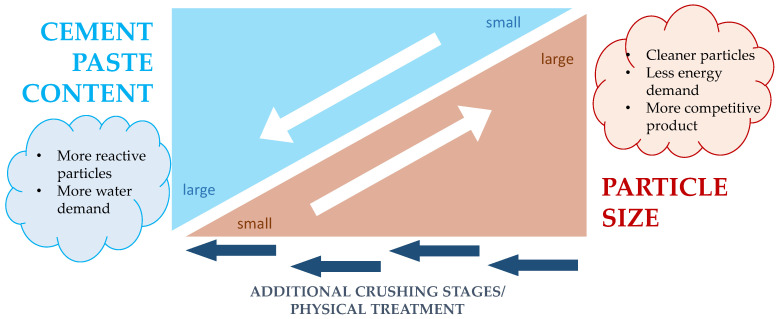
Impact of processing on RCA properties.

The crushing processes of RCA also influences the durable properties of RAC. Secondary crushing of RCA releases adhered mortar and reduces the porosity of RCA [39]. Overall, the crushing process and additional treatments shape the RCA particles and their features (Figure 3). When this low-absorption RCA is incorporated into concrete, it reduces the carbonation rate in comparison with single crushing RA [8]. The secondary crushing of RCA can reduce the accelerated kc of RAC by 9% to 18% when 100% RCA content is applied. In addition to the effect of reduced adhered mortar content, fewer planes of weakness remain [39] and a more rounded shape is achieved after secondary crushing. This last factor favors the workability of concrete and allows for reducing the mixing water and, eventually, the porosity of RAC.

Despite the improved features of RCA after secondary or tertiary crushing, the additional crushing cycles release the adhered mortar and reduce the relative volume of coarse RCA (cRCA) obtained from the waste concrete. Since the fine RCA (fRCA) fraction has lower valorization opportunities, at least at present, this increase in the quality of the cRCA reduces the competitiveness of the final product, and this is an aspect that still requires further analysis for practical application.

### 2.7. Enhancement Pretreatments to RCA

The features of the adhered mortar in RCA can also be improved by several strategies. Some treatments focus on removing adhered mortar (mechanically, similarly to secondary crushing, thermally, or chemically), while others strengthen the adhered mortar to reduce its porosity.

Some of the treatments to remove adhered mortar are mechanical grinding [40,41], ultrasonic bathing with a large amount of water [42], or partial dissolution in acid [43]. All these techniques proved technically effective to different degrees, but the main challenge remains in upscaling such technologies, especially to improve the cost–benefit ratio to produce competitive aggregates.

Alternative actions to strengthen the adhered mortar include mineral carbonation [44,45,46,47,48,49], applying polymeric emulsion [50] or pozzolan slurries [42,51], calcium carbonate biodeposition [52], or applying sodium silicate solution [45,53], among others. Again, these technologies increase the price of the material, and this increase is hardly justified by the improvement in properties. Additional discussion on such a cost–benefit analysis can be found in [54,55,56,57,58,59] Maybe mineral carbonation will differentiate from the others in the near future as the CO_2_ uptake has increasing potential for being monetized, at least in Europe with the implementation of negotiable carbon emission allowances.

At first, it can be thought that the full consumption of carbonatable phases in RCA with mineral carbonation reduces its buffer capacity and deteriorates the performance against carbonation of the RAC made with it. The reduction in porosity may be a prevailing physical effect over the chemical effect of reduced buffer capacity. Several authors [46,47,48,49,55,58,60,61,62,63] described the improved microstructure of mineral carbonated RCA. The reduction in porosity was confirmed in both the attached mortar and the old ITZ and reflected in an overall increased performance [54,64,65,66]. Specifically, improved carbonation performance was reported, with reductions of 28.68 and 45.33% in the carbonation depth at 28 [63,67] and 56 days [63,68], respectively, for 100% replacement of NA by carbonated RCA compared to the use of uncarbonated RCA. The evidence indicates that, with optimized parameters to reduce the porosity of the RCA, the improvement of the physical factor prevails over the detriment that may be caused by the reduction in the alkaline reserve. Moreover, the carbonation treatment should be emphasized in terms of the beneficial carbon uptake that it favors, saving carbon emissions by allowing the utilization of fume gases from the industry [69]. Very interesting reviews can be found in this regard in the literature [56,57,59,63,70,71,72,73], and for this reason, they are not extensively discussed in the present paper.

## 3. Particularities for Fine Recycled Concrete Aggregate (fRCA)

### 3.1. Influence of fRCA on Carbonation

Fine recycled concrete aggregates present more complex effects on the carbonation of concrete due to various factors. The shape of the fRCA particles can be determinant, as their angularity increases the paste demand for the same level of consistency [74]. Furthermore, the standard methods usually used to determine the water absorption capacity of sands are much less precise for fRCA [75], with a significant trend towards overestimating it. These characteristics make fRCA a quite complex constituent to be included in new cementitious mixes.

In [76], it was observed that concretes with 100% fRCA and coarse (cRCA) contents exhibited higher carbonation rates compared to concrete containing only cRCA. The carbonation rates can be increased up to 100% with the inclusion of fRCA. In general terms, the combined effects of fRCA and cRCA were comparable to the sum of the negative effects of the cases in which they were used separately. Evangelista and De Brito [77] found that with a 30% replacement, the carbonation rate was affected by the fRCA content, increasing by 40%. This result was not present in all the samples; some showed a surprisingly positive behavior, attributed to the chemical factor. As for non-cementitious CDW, such as fine recycled aggregate of ceramic origin, its inclusion is not recommended when carbonation resistance is required, as this is the property that is most negatively affected [7].

Geng and Sun [78] studied the influence of fRCA combined with fly ash (FA) in concrete mixes. The results indicated that as the particle size of fRCA decreases, the carbonation coefficient of concrete increases. For contents above 40% fRCA, conservative values for the *w/b* are recommended. A low FA content (below 20% of the binder) is also recommended, as it showed a positive effect on carbonation resistance.

An exception to the generally reported negative influences of fRCA is the study by Zega and Di Maio [79], who exposed natural carbonation concretes with fRCA replacement percentages of 20% and 30% and a *w/c* of 0.43. The carbonation rates were similar to those with 100% fine NA. This behavior was attributed to the moderately aggressive exposure environment, and additional studies seem convenient to reveal the extent to which accelerated carbonation tests are reliable to predict the carbonation of fRCA concrete (fRAC).

The quality of fRCA tends to decrease with an increasing number of reuses (second and third lives of RCA). However, the results of [80] showed a minimal impact on the performance of concrete incorporating fRAC involving one or two reuses. The difference in carbonation depth was only about 10% between fRAC after 1 reuse and fRAC after two reuses, with replacement ratios up to 40%. For 20% replacement, the lowest carbonation rates even occurred for the two recycling cycles.

### 3.2. Enhancement Pretreatments to fRCA

In recent years, research has focused on the carbonation and other durable properties of the attached mortar in fRCA. Shi et al. [67] evaluated clogging the attached mortar with pozzolanic slurries of silica fume (SF), nano-SiO2, and FA slurries. These treatments contributed to reducing the porosity and increasing the microhardness of the old ITZ at rates between 32 and 68%, varying with the type of aggregate, pretreatment intensity, and *w/b*. The carbonation coefficient of treated fRCA mortar demonstrated improvement relative to untreated fRCA mortar. The decrease in the carbonation coefficient after application of silica fume slurry treatment was 50% at an accelerated exposure of 14 days and 62.5% at an exposure of 28 days. This same trend occurred for other types of treatments, such as mineral carbonation of the fRCA, with a 42.5% lower maximum carbonation depth for concrete with treated fRCA (originated from mortar) compared to concrete with untreated fRCA [68]. Both types of treatment contribute to the improvement of the old ITZ. The authors have attributed these results to the reduction or clogging of the porosity of the attached mortar as the predominant factor.

## 4. Combined Action with Supplementary Cementitious Materials (SCM)

The use of recycled aggregates in combination with supplementary cementitious materials (SCM) is a trend that is gaining momentum in the quest to reduce the global carbon footprint. The use of SCMs in RAC amplifies the sustainability and durability of the material thanks to the pozzolanic action. However, this pozzolanic action also consumes portlandite, and with higher SCM contents and low clinker contents, the carbonation rate can increase and also lead to decalcification of the C-S-H [19]. The integrity of C-S-H is associated with a sufficient alkaline reserve. If the alkaline reserve is limited, not only does the pH drop due to the carbonation, but pore coarsening may also occur [81]. This is actually contrary to the porosity reduction in RCA achieved with mineral carbonation and deserves particular attention for RCA coming from concrete with a low clinker content.

Seeking a combination of RCA and SCM that does not dramatically affect portlandite formation is a valid alternative to counteract carbonation. In [82,83], it was reported that a combination of SCMs such as 20% FA and 10% metakaolin (MK) partially replacing CEM contributes to improving carbonation resistance for 25% RCA content. On the other hand, 10% SF completely nullifies the negative effect of RCA with respect to the carbonation of the reference concrete, whether it is fRCA or cRCA, and at replacement levels of NA by RCA below 50%. A comprehensive review and analysis of the carbonation of concretes with various SCM can be found in [81], highlighting the disadvantageous performance in those cases in which significant portlandite consumption does not lead to appropriate microstructure development.

Fly ash (FA) has emerged as one of the most thoroughly studied SCMs in combination with RCA. Kurda et al. [76] reported accelerated carbonation of concrete with 100% fRCA and cRCA by a factor of up to 2.7 times with respect to the reference concrete with NA and CEM I. The replacement of CEM I by FA at ratios of 30% caused a similar effect with a factor of 3 times the increase in carbonation depth. On this basis, concrete cover would need to be increased by 40 or 200% for respective FA contents of 35 or 40–70% [84]. FA is well known for decreasing the carbonation resistance of concrete due to a combination of reduced reactivity and the consumption of portlandite. Other similar SCMs (e.g., sugar cane bagasse ash, even silica fume) may have a similar effect [85]. Interestingly, the combined action of fRCA and FA resulted in an increase in the carbonation rate that was lower than the sum of the separate individual actions of fRCA and FA. Whereas the effect of fRCA is primarily physical (connected to the porosity of the aggregate), the effect of FA is primarily chemical (connected to the portlandite reduction). This combination could imply mutual compensation between the effects of fRCA and FA. In [86], various replacement percentages were studied for diverse strength classes. In agreement with the previous study, there was an increase in carbonation rate with the inclusion of both FA and RCA. Interestingly, for a strength class of C35 or higher, there was a marked reduction in the difference between RAC and NAC over time up to 20 weeks of age. This can be explained by a similar pozzolanic action of FA both in RAC and NAC and the fact that high RCA contents may provide additional alkaline reserves [12,16], favoring the reaction of FA and clogging effects due to carbonation even more. As for natural carbonation, results are reported for concretes with up to 100% cRCA and up to 55% FA [6]. After 10 years of exposure, the dilution effect of FA resulted in increasing factors for the carbonation rate of approximately 1.68 and 1.89 times for unblended NAC and RAC, respectively. In this case, the combined action was greater than the sum of the separate effects, revealing notable differences between predictions based on accelerated tests and results from natural exposure. In general terms, the performance of FA concrete concerning carbonation should be weighed against the reduction in environmental impact achieved by using reduced clinker factors, and this balance is in general beneficial for FA concrete [87].

Very reactive SCMs can have a different impact on the carbonation of RAC than that of FA. For example, Pedro et al. [88] studied the combined actions of SF and FA together with cRCA and fRCA. They found a decrease in the carbonation coefficient at 91 days of approximately 40% with the inclusion of SF (5–10%) in both control and RAC. In this case, the prevailing effect of the SCM is the densification and reduced permeability of concrete. The fact that RCA was subjected to secondary crushing must be taken into account. The presence of SF also lessened the negative influence of the RCA, offering more similar results in RAC with respect to NAC. A similar but more moderate contribution from GGBFS was described in [89,90,91], where the carbonation rate of an RAC was reduced (about 15–25%) with the incorporation of low contents of GGBFS. In the case of GGBFS, the limited portlandite consumption of this SCM for developing microstructure seems to be an advantage over other SCMs concerning carbonation. The relationship between the physical pozzolanic action and its capacity to counteract the chemical pozzolanic action has great relevance in terms of the net action of each SCM on carbonation resistance.

## 5. Discussion

Most of the works consulted highlight the higher porosity that RCA provides to RAC due to the content of adhered mortar and the presence of weak old ITZ. As could be verified, the hardness of the old ITZ can be very low, and this has consequences for the transport properties of RAC.

In order to counteract these consequences, removing or improving the attached mortar is the main recommendation. Secondary crushing is one of the most widely used methods for such aggregate upgrading. During crushing, the result may not be successful in improving the carbonation rate if microcracking occurs. The effectiveness of the process depends on the crushing method used and the original aggregates. Secondary crushing also produces a greater amount of fRCA in relation to the total volume of waste concrete, and this reduces the competitiveness of the secondary products of the whole process. Unless high-value uses for the fRCA are found, the implementation of attached mortar removal at an industrial scale will be limited by the cost–benefit ratio. An alternative form of mitigation that has shown good results is the mineral carbonation of RCA with CO_2_ after its use in RAC. The issue of treatment costs is also present here, but with the advantage of constituting a carbon capture technology that can be monetized.

A counteracting and important factor to that of increased porosity is the content of residual portlandite that this same attached mortar in the RCA provides. A good quality of the attached mortar with sufficient portlandite content may make it possible to avoid the need to remove this mortar. In this respect, a threshold RCA content is a key point of attention in the prediction of carbonation depth, which is defined by the ratio of porosity to buffer capacity of the RCA, its content, and the *w/c* of the new mix. This explains to some extent the disagreement sometimes found in the literature, highlighting the fact that RCA of different levels of quality bring different outcomes in terms of carbonation, and allow RCA contents of even 70% without significant impact on the carbonation rate.

The porosity of RCA has potential additional indirect effects on the new matrix. The effective water/cement ratio can differ from the total water/cement ratio if RCA develops active water uptake in the fresh mix. Thus, this water uptake derived from the porosity of the aggregate should be calculated as accurately as possible, taking into account the water absorption and the initial moisture state of the RCA. It is also important to consider that the experimental water absorption of the aggregate as a constituent is generally higher than the estimated water uptake from the effects on the consistency of concrete. Whenever the mixing water is compensated for the full water absorption, increases in slump are normally reported [92,93]. Another point of attention for the saturation state of RCA is the fact that saturated RCA incorporation lowers the quality of the new ITZ, facilitating transport such as CO_2_ diffusion through this weaker phase.

Furthermore, the quality of the new ITZ also depends on the pre-processing of the RCA. With mortar removal processing, the surface of particles may become harder and develop a better bond and less porous ITZ thanks to the removal of weakly attached material. Additionally, strengthening the adhered mortar may contribute to the strengthening of the new ITZ as well as the old ITZ, but normally to same or lower extent than the mortar removal. Strengthening has the advantage that it does not reduce the portlandite content.

The impact of the type and contents of cementitious materials in concrete is partially derived from their interaction with the RCA. The increase in permeability caused by RCA combined with either the clinker dilutive effect of low reactive SCM or the densifying effect of high reactive SCM are highlighted as the most relevant physical aspects. The dilution and consumption of the portlandite content can be partially compensated by the incorporation of RA. There would normally be more portlandite in fRCA than in cRCA, due to the more adhered mortar content in the fines. This would depend on the storage history, as finer particles would carbonate much faster prior to use in new concrete mixes. At the same time, the pozzolanic action of SCM could partially compensate for the additional porosity provided by RCA. This is an aspect that deserves further research, especially in relation to particle size.

This means that the influence of RCA particle size on carbonation performance does not only relate to the size itself but also to composition. Decreasing particle size implies an increased amount of adhered mortar. The extent of the real effect in concrete mixes is masked by the various (and sometimes incorrect) compensation methods for the water uptake of fRCA. An increased carbonation rate at various replacement percentages with fRCA is generally reported, but there is no consensus on its quantitative impact. For high replacement percentages, the adhered paste content may provide a larger alkaline reserve, but increased carbonation resistance has not been proven. Under normal conditions, fRCA showed more room for improvement through pretreatments than cRCA (e.g., more carbonatable material for mineral carbonation), but the commercial feasibility of such treatments is still an unresolved issue. In particular, the influence of fRCA on the carbonation of concrete has not been completely resolved, and for this reason, a number of investigations in this field are still necessary.

**Figure 4:**
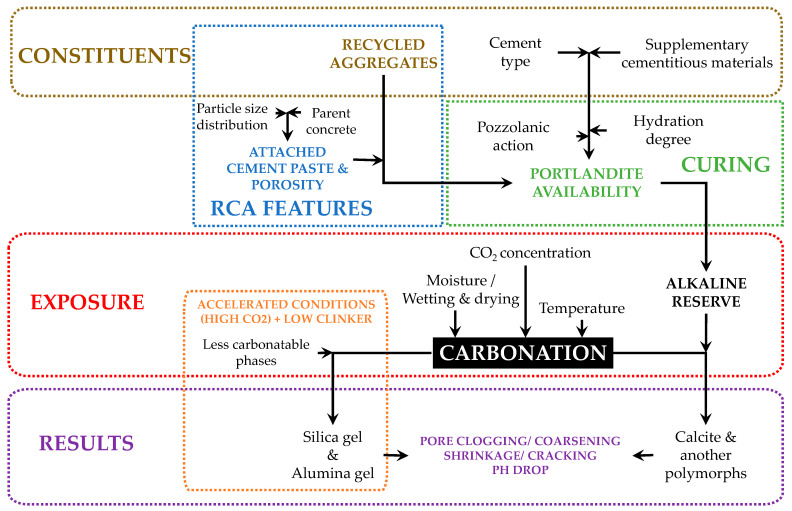
Process sequence towards carbonation and its effects.

Conversely, mineral carbonation is not only interesting for the improvement of the physical properties of the aggregate. Perhaps even more interesting in the case of fRCA is the carbon utilization capacity to reduce emissions from the industry. The pretreatment of RCA favors their carbonation prior to use, which is greatly impeded once the particles are embedded in a new cementitious matrix. Even when already in service, finding a balance between preventing carbonation to secure the durability of reinforced concrete and favoring it to capture CO_2_ is a challenge. The second option is very clear in the case of non-reinforced concrete, in which no risk of corrosion is present. Still, very low clinker factors imply a limited amount of portlandite that can allow increased decalcification of C-S-H with potential coarsening of the pore structure of the matrix. The role that RCA can play in this sense is interesting to the extent that it contributes to the overall alkaline reserve. The competition for portlandite between reactive SCMs and carbonation can be mitigated with the use of RCA. The process sequence towards carbonation and contributing processes previously discussed are summarized in Figure 4.

**Figure 5:**
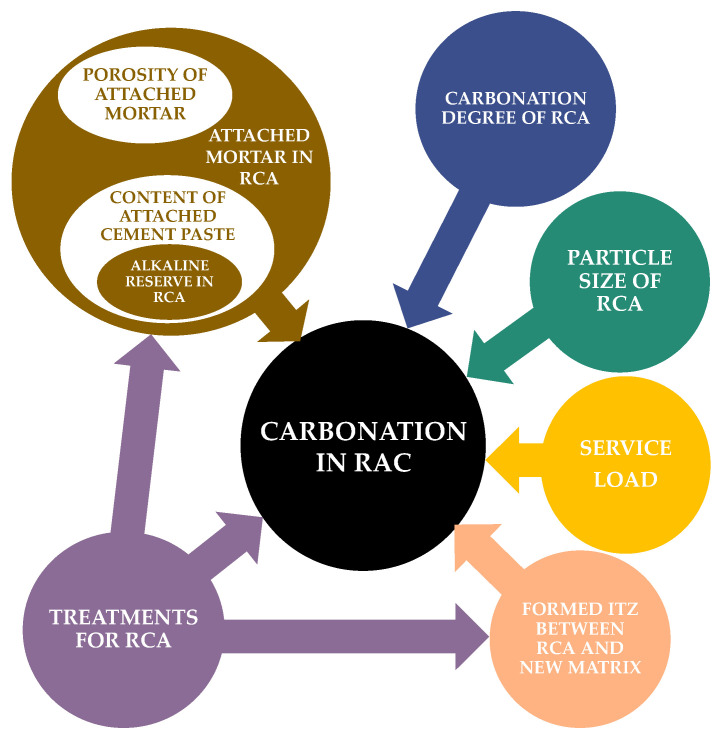
Factors affecting carbonation of RAC.

Therefore, the prediction of the carbonation of RAC involves a multitude of factors. The importance or weight of each factor is closely tied to the characteristics of each specific RCA. Generally, these factors can be considered in a certain priority order, as depicted in Figure 5. The prediction process involves not only assessing the carbonation rate (the speed at which the carbonation front progresses), but also the carbonation degree. For RAC, as for any other type of concrete, precise estimation of the degree of carbonation is still a matter of debate, as it has been shown that natural carbonation in service may deviate from the results of accelerated tests. Moreover, the complexity of the variables (from the material and from the exposure environment) together with their interactions should be considered within a probabilistic approach [94]. The prediction of RAC carbonation is even more complex than for NAC due to these additional aspects.

The porosity of concrete also receives contributions from microcracking as a determining factor for carbonation. This level of microcracking depends not only on the crushing of the RCA but also on the levels of stress to which the RAC is subjected in service. Low compressive loads generally decelerate carbonation, while high compressive loads or tensile loads accelerate carbonation. For compression, if the threshold for the stress level is exceeded, the microcracking deteriorates the integrity of the concrete, allowing additional paths for the ingress of external substances such as CO_2_ and water. The effect of loads, with particular consideration of RAC features that may make them more susceptible to creep, is another complex effect that requires additional research for its modeling.

## 6. Conclusions

The main factors influencing the rate of carbonation of RAC and the recent advances in our knowledge of the phenomenon are discussed in this article. The higher complexity in comparison with NAC and the need for increased circularity and sustainability in the concrete industry make this a hot topic for research. Future studies on this topic are obviously very necessary, and the following conclusions aim to contribute to them:

1. The carbonation of RAC depends on a large number of variables. The most important aspects are related to the porosity of the adhered mortar and the original natural aggregate in waste concrete. Additionally, differences arise in terms of composition depending on the particle size, with more adhered mortar in fine particles than in coarse particles. Progress must be made in the understanding of the factors that determine the degree of carbonation and in the physical and physical-chemical influencing aspects of the RCA and the fRCA.
2. The adhered mortar in RCA is the most influential factor concerning the carbonation of concrete made with it. It has a dual impact: it affects the porosity with a derived faster diffusion of CO_2_ and the alkaline reserve. As such, this attached mortar is the main actor in the mineralization of CO_2_ in RCA pretreatments. The effect of the attached mortar is also, in the second term, associated with the characteristics of old and new ITZs that are formed in new concrete. Low-quality adhered mortar forms weak ITZ with NA and favors even more CO_2_ diffusion. Secondary crushing for removing the adhered mortar is another enhancement treatment that contributes to the improvement of the old ITZ (as long as no significant additional microcracking is produced), but it reduces the production ratio of cRCA relative to original waste concrete and the contribution of the RCA to the alkaline reserve.
3. The feasibility of post-processing RCA to improve their properties is technically proven. However, large-scale feasibility is more doubtful in terms of producing competitive products. For example, cement slurries can strengthen the new ITZ, but the consumption of cement or SCMs increases the cost and environmental impact of the product. Other improvement alternatives, such as treatment with CO_2_ at high pressure, have proven more advantageous in terms of sustainability but are still relatively expensive compared to the production of NA. The reduction in the alkaline reserve, especially in combination with the use of SCM (in the adhered mortar or in the new matrix), is a matter of high research interest to address the competition between the pozzolanic reaction and the carbonation reaction.
4. Regarding the contribution of RCA to the alkaline reserve, a significant influence has been found at high substitution percentages (between 70% and 100% of cRCA). For fRCA, this influence seems somehow limited by the carbonation prior to use. The content of adhered mortar proved to lead to high success for contents higher than 40% and should always be analyzed. In the case of high percentages, this alkaline reserve is connected to the negative physical properties of the adhered mortar, and despite showing a positive impact on carbon uptake, it is possible that the same is not true for the carbonation rate.
5. The phenomenon of microcracking due to the application of service loads also affects carbonation. This is true not only for RAC but for all types of concrete, but the special features of RAC may increase creep. In general, moderate compressive stresses improve the carbonation resistance, and as the load level approaches the maximum strength of the concrete, it will degrade the carbonation resistance. Tensile stresses always reduce carbonation resistance.
6. Balancing strategies are essential to achieving sustainability in the concrete industry. Reducing the carbonation rate in reinforced concrete and capturing as much CO_2_ as possible in non-reinforced concrete is a good compromise for fit-to-purpose design. Still, it must be considered that very low clinker factors in the binder may still lead to decalcification of C-S-H, raising some concerns about the benefits of carbonation in non-reinforced concrete. When RCAs are paired with reactive SCM, adequate performance will be obtained as long as densification of the cementitious matrix is achieved, with the potential advantage of RCA to mitigate the decalcification of C-S-H in low-clinker concrete.

## Data Availability

No new data were created for this publication.
